# On the Odours Which Are Exhaled by Living Animals

**Published:** 1801-11

**Authors:** J. J. Virey


### *On the Odours which are exhaled by living Animals.* *By* J. J. VIREY.

[ Concluded from p. 346, of our last. ]

THE insupportable stench which is emitted by some animals belonging to the family of genets, seems to be of the same kind as the former, (castoreum,) but only exalted and mixed with the hydrosulphureous smell, and that of other hydrogenous gases. It serves those animals for the purpose of preventing their enemies pursuing them. The *Viverra nasua*, (1.) *Screb. Viverra vulpevula*, *L.* (2.) *V. putorius*, *L.* (3.) (pole-cat,) *V. mephitis*, *L.* (4.) and several others are known to cast that offensive smell, and likewise the glutton, (5.) the polecat, (*Mustela putorius*, *L.*) and the fox, &c. emit their bad odours against the dogs that pursue them. On the death of the animal these odours are considerably diminished, because they cease to be secreted; they adhere however so strictly to any substance which is impregnated by them, that it is very difficult to deprive it of the smell entirely by washing; for which purpose, therefore, KUHN advises to put it under ground for twenty-four hours. The hyæna, *Canis hyæna*, *L.* the *Canis*

---

(1.) SCHREBER Saeugthiere (Mammalia,) tom. iii. p. 436. MARCGRAF Histor. Brasiliæ, p. 228.

(2.) YZQUIEPATT of Hernandez Hist. Nat. Mexic. p. 332; and likewise the Viverra quasje, SEBA Mus. Thesaur. tom. i. p. 66, 68.

(3.) *Polecat* of CATESBY, Nat. Hist. of Carolina, tom. ii. p. 62. KALM Amer. tom. ii. p. 378.

(4.) PENNANT's Synopsis of Quadrupeds, p. 233, no. 167. BUFFON, l. c. xiii. p. 294.

(5.) SCHOEFFER Historia Lappon. p. 339. GUNNER Act. Nidros. tom. iii. p. 123, tab. 3, fig. 7. GENBERG Act. Stockholm, 1773, p. 222, tab. 7.

*crocuta*, *L.* (1.) and the *Canis corsac*, ſpread alſo a very diſagreeable odour, which however ſeems to be peculiar to the whole family of Mammalia, and of carnivorous birds, and their breath is ſo bad in general, that is has been ſaid to produce pulmonary conſumption in people which inſpire it, eſpecially that of the cat.

The ſtrong odour of the bats, and even of the owls, comes very near to that of the former animals, but it has ſomething nauſeous in it, which has been obſerved to occaſion ſyncope in ſeveral perſons. The ſmell of ſeveral ſerpents is very analogous to it; and, according to KALM, the cauſe of that pretended faſcination by which they are ſuppoſed to attract or rather ſtupify the little animals, depends on that vapour, by which they are, as it were, ſuffocated. It is alſo known that the odour of the *Aplysia depilans*, *L.* (2.) or lepus marinus of the ancients, is very diſagreeable and nauſeous.

Amongſt the birds, the family of the *coraces* exhibits a peculiar ſmell, and amongſt the vultures, the *Vultur jota* of Molina, (3) is particularly remarkable for its odour, which is like to excrements; and JACQUIN ſays, that this bird frequently caſts up ſtuff of a ſtill more fetid ſmell. In general, the odours of the greateſt part of carnivorous animals ſeem to be owing to the putrid animal matters on which they feed.

All animals of ſweet water, as well as of the ſea, are endued with a peculiar odour, which is eaſily diſtinguiſhed from all others, particularly in the rutting ſeaſon, but it evolves itſelf ſtill more when they begin to be expoſed to putrefaction; the fetid ſmell of the ſtinking orach, *Chenopodium vulvaria*, *L.* has a great analogy to the former odour, which is particularly ſtrong in the carnivorous fiſhes. It has been thought that this fetid ſmell has its ſeat only in the ſkin of thoſe animals, but it appears to me that their fat is not free from it, beſides the rancid odour which is frequently perceived in it at the ſame time.

Rancidity, which is a ſeparation of ſebacic acid and phoſphoriſed or carboniſed hydrogen gas in oily ſubſtances, particularly thoſe of marine animals, has a great influence on the odours of thoſe animals that live upon them, ſuch as the ſea birds, and the ichthyophagous nations which inhabit the ſhores of the nothern ſeas. Theſe men, and eſpecially theſe birds, as the *Colymbi*

(1.) LUDOLF Hiſt. Æthiopiæ, lib. i. c, 10. ERXLEBAN Mamm. Hiſt. p. 578.

(2.) BOHADSH de Lernća, tom. iii. p. 51. RONDELET de Piſcibus, tom. i. p. 519.

(3.) Hiſtor. Natur. de Chili, p. 235.

*Colymbi* of LINNÆUS, the penguins, gulls, the pelecani, &c. and all thofe which are in general comprifed under the name of *Anseres*, have a very rancid flefh, which difagreeable tafte is even communicated to their eggs; it is likewife found in thofe turtles that feed on animal matters, as the Teftudo caretta, *(the hawks-bill turtle,)* (1.) &c.

The immenfe tribe of infects, prefents feveral fpecies which fpread very fetid exhalations, fuch as the *Staphylini*, *Carabi*, *Sylphæ*, *Coccinellæ*, *&c.* all which emit a very ftinking liquor, when they are caught, and likewife the caterpillar of *Bombix lossus*, Fabric.

The *Hemerobius perla*, *L.* impregnates every thing which it touches with the fmell of excrements; the fmell of the *Reduvius acanthiæ*, and of many other fpecies of bugs, is alfo very offenfive, and is likewife found in that umbelliferous plant which furnifhes the coriander feed. The *Scarabæi*, *Spheridiæ*, &c. fmell of the putrid matters in which they live.

All odours, whether good or bad, which are exhaled by animals with red blood and vertebræ, derive for the moft part their origin from a ftratum of glands, fituated in almoft all the mammalia, (2.) and in the reptiles, (3.) about the anus, and on the rump, (coccyx) of birds. In fome quadrupeds, however, they are placed near the organs of generation, with which they feem, according to GMELIN's opinion, (4.) to be all connected; in the human fpecie they exift likewife near that place, under the form of miliary glands. The fmelling vapours fecreted by thofe glands, appear moft probably to be intended by Nature, for attracting the two fexes to the act of generation, becaufe they are particularly evolved in the rutting feafon, and begin to diminifh confiderably when this ftate of orgafm has ceafed; very odorous animals therefore have been found to be particularly hot in love. In fome animals however, the odours have the function of defending them againft their enemies. The exhalations in the family of reptiles are of a particular kind, being not only very difagreeable and alliaceous, as thofe of the toad and falamander, which come near to the fmell of *assafœtida*, but alfo fometimes fo much concentrated, that they have been fuppofed to caufe death, a circumftance which is related by MARTIN DELRIO, in his Magic Difquifitions of the Vapour of irritated and angry Toads and Vipers.

(1.) BROWN's Hiftory of Jamaica, p. 465, no. 3.

(2.) DUVERNEY Oeuvres Pofthumes, tom. ii. p. 559.

(3.) CHARAS de la Vipere, tab. ii. TYSON Philofoph. Tranfact. no. 144. SEVERINUS de Viperibus, p. 236.

(4.) Nov. Comment. Acad. Petropol. tom. iv. p. 399.

pers. (1.) The exhalations of the *Lacerta geoko*, (2.) and *L. Geitje* (3.) are dangerous, even the touching these animals is thought to produce violent inflammations and excoriations.

Although I have endeavoured to multiply the classes of animal odours as much as possible, yet there are some odours which it is impossible to refer to any of them, and I never could unite the smell of *ambergris*, *assafœtida*, and musk, with that of opium and of bugs, as has been done by LORRY. (4.) The celebrated LINNÆUS has divided the different odours of medicines into the following classes: 1. *Aromatici.* 2. *Fragrantes*; 3. *Ambrosiaci*; 4. *Alliacei*; 5. *Hircini*; 6. *Tetri*; 7. *Nauseosi*; (5.) a division which appears to be satisfactory with respect to the vegetable kingdom, of which it is made; but the great HALLER very justly remarks, that a great number of animal as well as chemical exhalations, cannot yet exactly be ranked after a certain order, (6.) such as the resinous odours of elemi, &c. the farinaceous which are perceived in some mushrooms, the odours of several species of *Geranium*, *Aristolochia*, *Iris*, *Diosma*, *Herculia*, *Galium*, *Asperula*, of green figs, the grasses and orchideæ, the jalappa, the bituminous exhalation of a *Psoralea*, the odour of some papilionaceous flowers, the aromatic scent of coffee and of roasted corn, the strong smell of *Myrica gale* and *Ledum palustre*, the sweet exhalations of Alpine plants, the smell of fucusses, and many others which we cannot exactly determine. The kinds of odours vary so much in general in the vegetable as well as animal creation, that it is a vain attempt to reduce them all to six or seven classes. This variety of odours even extends itself to the human species, the different nations of which seem to exhale an odour which is peculiar to them. It is known that the negroes of certain countries in Africa, such as the Jotofs, exhale a fetid odour when they are hot, similar to that of porret. The Samoiedes, Esquimaux, and other northern nations have a peculiar smell, which however, seems to depend on their nourishment, which mostly consists of half rotten animal food. (7.)

It

(1.) BOERHAAVE Prælection. Instit. tom. iv. p. 77.

(2.) HOUTTUYN Acta Vlissing. tom. ix. p. 322. LAURENT Amphib. p. 44, no. 57. HASSELQUIST's Tour to Palest. p. 306.

(3.) ANDREW SPARRMANN Act. Gothenburg. vol. i. p. 75.

(4.) Memoires de la Société de Medicine, extract. by HALLE, vol. v. 1785, p. 307, 308.

(5.) Amoenitates Academicæ, vol. iii. art. 38, by Wahlin, 1752, (ed. Schreber.)

(6.) Elementa Physiologia, lib. xiv. sect. ii. § 5, p. 162, vol. v.

(7.) KLINGSTÆDTT Memoires sur les Samoiedes, p. 36. COR-

NEILLE

It is likewife known, that the fouthern Europeans frequently fpread an alliaceous odour, becaufe they are accuftomed to eat the bulbs and roots of this family of vegetables. Perfons that live on nothing but vegetables, have a fweat that fmells acid, which is alfo the cafe of little infants. People with red hair exhale a very ftrong odour, particularly arifing from the arm-pits. (1.) The exhalations of perfons of a dark complexion, and of thofe who are very hairy, are rather alkaline, and fomewhat analogous to the ftrong fcented breath of carnivorous quadrupeds; of this kind are the mongolic fpecies of men, and thofe of bilious conftitutions, as it is commonly called. The ingenious BORDEU however remarks, that it may be confidered on the whole as a proof of vigour and force, particularly in the genital fyftem.

The accurate examination of the different odours may prove very ufeful for explaining feveral phenomena of the difeafed ftate of the human body, a fubject which has been already fuccefsfully treated by *Bordeu* and *Brieude*. (2.) Several difeafes exhibit peculiar odours, fuch as the acid and difagreeable odour of fome miliary fevers, (3.) the infupportable ftench that we perceive in the jail fever, (4.) and in the bubo peftilentialis. (5.) It is known, that odours have been employed fince the moft remote ages as medicines, a practice attributed by AETIUS (6.) to CRITON, a phyfician much older than GALEN, and ufed alfo by later phyficians, as John Bravus. (7.) JOHN VICTOR CONRAD SCHNEIDER mentions this fubject in his excellent treatife *De osse cribriformi*. Modern phyficians have likewife paid a particular attention to it, (8.) as of late Mr. ALIBERT.

Although the hiftory of Democritus, who lived during three days on the vapour of hot bread, and the *olfactive aliments* of Hippocrates,

---

NEILLE DE BRUYN, Voyage II. vol. i. Voyages au Nord, vol. ii. PAW Recherches Philofophiques fur les Americains, vol. i. p. 218. O. FABRICIUS Fauna Groenlandie, clafs i. ANDERSON's Ifland, vol. i. Egede Hiftor. Groenland. I. fect. 2. ERIC. PONTOPPIDAN's Hiftory of Norway, part ii. OLAUS MAGNUS Degentibus Septents. lib. iv. PENNANT's Tour to the Hebrides, vol. ii. ANSON's Voyages, &c.

(1.) LORRY Morbi Cutanei, fect. ii.

(2.) Analyfe Medic. du Sang, &c.—BRIEUDE Memoires Societ. Med. Year 1789, p. 45, &c.

(3.) GRAINGER de Febr. Batav. p. 33.

(4.) PRINGLE's Difeafes of the Army, p. 310.

(5.) SYDENHAM Oper. p. 174.—Lieutaud Precis de Medicine, p. 147.

(6.) Tetrabibl. ii. ferm. iv. c. 7.

(7.) De Sapor. et Odorum Differentiis, Caufis et Effectibus. Salamanticæ, 1513, 4to.

(8.) AUGUSTIN CIGARINI Novæ de Odoribus Theoriae Trutina. Senis, 1749, in 4to.—J. CORVIN de Organfens, et Objecto Olfactus. Prag. 1749, in 4to. &c.

Hippocrates, are ſufficiently known, yet it is difficult to believe that theſe exhalations ſhould immediately act on the organs of nutrition; there is, however, no doubt of the exhalations of certain medicines having occaſioned purging in ſome people, a fact which is proved by experience: The nauſeous odours frequently produce vomiting, all which ſhows a ſtrict ſympathy betwixt the ſtomach and inteſtines, and the ſenſe of ſmelling. It ſerves, therefore, to animals in the choice of their food, and ſeems to be formed, and, as it were, adapted to the nutrition of each reſpective animal, none of which can be without that ſenſe, ſo powerful in animal œconomy.

With regard to the medical uſe of odours, it has been obſerved, that they are particularly efficacious and ſerviceable in all nervous affections; the fetid animal exhalations, for inſtance, in epileptic paroxyſms; the ammoniacal ones in lipothymies, &c. But in order to enquire into the particular utility of odours in medicine, it is neceſſary to divide them into claſſes, which poſſeſs very different properties. In general it may be obſerved, that the agreeable odours are not ſo dangerous as the nauſeous ones.(1.)

The fragrant and ſomewhat narcotic odours of ſpring flowers are very uſeful in aſſuaging the too irritable conſtitution; whereas, the nauſeous odours never agree with them. The aromatic ſmells are tonic and excitant, and likewiſe the muſky, which are particularly aphrodiſiacal; they exhauſt, however, by degrees, the excitability, and produce a kind of ſtupor, and conſiderable debility. Very volatile and odorous remedies have been obſerved to do harm to ſcorbutic, as well as hypochondriacal perſons, whoſe ſmell is in general, very ſenſible, and delicate.

Camphorated exhalations frequently produce head-achs, particularly to nervous women. Odours are ſaid to diminiſh the vital power, (2.) but ſometimes they produce a kind of drunkenneſs, which I ſaw in a young lady, accompanied with a ſlight delirium.

It ſeems that the nauſeous odours exhale a great deal of carbonic acid, whereas the more volatile odours diſcharge in general hydrogen gas, impregnated with a certain quantity of volatile oil, which is different in each ſpecies of the above exhalations.

There ſeems to be a difference in the ſcents of the different nations, becauſe the ſtrong narcotic odours are better liked by the

(1.) Linne Philos. Botan. p. 285.

(2.) Lorry Mem. Soc. de Medic.

the fouthern nations, and the ethereal exhalations are more efteemed by northern people; it has even been obferved, that fome nations have no perception for a certain odour, when, on the other fide, their fmell is extremely delicate for a different exhalation. Thus, the people of Kamfkatcha did not fmell the fcent of *aqua melissæ spirituosa*, but they difcovered at a great diftance, by the fenfe of fmell, a rotten fifh, or a ftranded whale. (1.) The power of fmelling depends, in a great meafure, on habit and cuftom; and the men who live in focial life, are more affected by vegetable odours, while the favage fmells better the putrid and fetid exhalations of animal bodies. (2.)

In general, each animal feems to emit a peculiar exhalation, which is more or lefs ftrong in fingle individuals, according to their refpective character and temper. There is, however, a great analogy of odours in fome families, orders, or claffes of animals, fo that it requires confiderable practice to diftinguifh the difference.

(1.) Second Voyage of Captain Cook, Vol. IV.

(2.) Virey Effai d'Hiftoire Naturelle et Phyfiolog. fur la Perfectibilité de l'Homme. Paris, in 8vo.

 ftimulated

